# Food environment of bus terminals in the Rio de Janeiro metropolitan region

**DOI:** 10.11606/s1518-8787.2024058004769

**Published:** 2024-02-09

**Authors:** Ana Carolina Castro de Jesus, Laís Vargas Botelho, Daniela Silva Canella, Letícia Ferreira Tavares, Paulo César Pereira de Castro, Isabela da Costa Gaspar da Silva, Letícia de Oliveira Cardoso

**Affiliations:** I Fundação Oswaldo Cruz Escola Nacional de Saúde Pública Sérgio Arouca Programa de Pós-graduação em Epidemiologia em Saúde Pública Rio de Janeiro RJ Brazil Fundação Oswaldo Cruz. Escola Nacional de Saúde Pública Sérgio Arouca. Programa de Pós-graduação em Epidemiologia em Saúde Pública. Rio de Janeiro, RJ, Brazil.; II Universidade do Estado do Rio de Janeiro Instituto de Nutrição Departamento de Nutrição Aplicada Rio de Janeiro RJ Brazil Universidade do Estado do Rio de Janeiro. Instituto de Nutrição. Departamento de Nutrição Aplicada. Rio de Janeiro, RJ, Brazil.; III Universidade Federal do Rio de Janeiro Instituto de Nutrição Josué de Castro Departamento de Gastronomia Rio de Janeiro RJ Brazil Universidade Federal do Rio de Janeiro. Instituto de Nutrição Josué de Castro. Departamento de Gastronomia. Rio de Janeiro, RJ, Brazil.; IV Universidade Federal do Rio de Janeiro Instituto de Nutrição Josué de Castro Departamento de Nutrição Social e Aplicada Rio de Janeiro RJ Brazil Universidade Federal do Rio de Janeiro. Instituto de Nutrição Josué de Castro. Departamento de Nutrição Social e Aplicada. Rio de Janeiro, RJ, Brazil.; V Fundação Oswaldo Cruz Escola Nacional de Saúde Pública Sérgio Arouca Departamento de Epidemiologia e Métodos Quantitativos Rio de Janeiro RJ Brazil Fundação Oswaldo Cruz. Escola Nacional de Saúde Pública Sérgio Arouca. Departamento de Epidemiologia e Métodos Quantitativos. Rio de Janeiro, RJ, Brazil.

**Keywords:** Food, Health of the Urban Population, Means of Transportation

## Abstract

**PURPOSE:**

To describe and analyze the healthiness of formal and informal food establishments in bus terminals of the metropolitan region of the state of Rio de Janeiro.

**METHOD:**

An audit was conducted in 156 formal and 127 informal food establishments located in 14 bus terminals of the five most populous cities of the metropolitan region of Rio de Janeiro. Proportions of types of establishments and means (95%CI) of food availability indicators in formal and informal settings were calculated. For the formal setting, prices, proportions of accepted payment methods, days and hours of operation, and food categories with displayed advertising were described.

**RESULTS:**

The healthiness of food establishments in bus terminals was low (less than 36%). On average, ultra-processed food subgroups were 250% more available for purchase than fresh or minimally processed food. Purchasing food at these places was convenient because several forms of payment were available, and the opening hours of the establishments followed the peaks of movement. In addition, 73.3% of the advertising referred to ultra-processed drinks, and the cost-benefit of buying ultra-processed food was better than fresh or minimally processed food.

**CONCLUSION:**

The food environment of bus terminals in the metropolitan region of Rio de Janeiro promotes unhealthy eating. Regulatory public policies should focus on initiatives to limit the wide availability and advertising of ultra-processed food in spaces of great circulation of people.

## INTRODUCTION

Bus terminals are strategic connection points between intra- and inter-city bus lines. Buses are the most used means of public transportation in Brazilian metropolitan regions, including the metropolitan region of Rio de Janeiro (MRRJ)^1^, which is the second largest in Brazil and fourth largest in Latin America, with an estimated population of 12.8 million inhabitants in 2019^2^.

In the municipality of Rio de Janeiro alone, 1,008,326,226 bus boardings were recorded in 2019^3^. Despite their importance, urban mobility systems are inefficient and travel time in large Brazilian urban centers is high, especially in RMRJ, with an average of 67 minutes^4^.

Studies conducted in high-income countries have shown that a significant portion of eating away from home is done on the way from home to work or study^5^ and that food and drinks available on public transportation have low nutritional quality^6^. Evidence from low/middle-income countries agrees on the low nutritional quality of the items sold, but studies are scarce and focus mainly on food availability^7,8^. Although the body of evidence on how the built space of cities, green areas, air pollution, and even the food environment impact individual behaviors is not recent^9^, there are still gaps in the characterization of the food environment of public transport facilities in Latin American metropolises.

The food environment can be understood as the “physical, economic, policy and sociocultural surroundings, opportunities and conditions that influence people’s food and beverage choices and nutritional status”^10^. In addition to the availability of food and drinks, there are other important dimensions to understand the relationship of the food environment with eating practices: affordability, accommodation^11^ and food and drink promotion^12^.

Studies on the food trade in public transport facilities prioritize formal^6,8^ over informal^7^ sales. The informal food setting is one not regulated by formal governance structures, such as kiosks and street vendors^13^. Informal vendors represent a significant share of the food supply in low- and middle-income countries, especially among people of low socioeconomic status^14,15^. Informal food and drink vending is a common practice on public transportation in Rio de Janeiro^16^.

Despite the intense use of public transportation^3^, high average travel time in large Brazilian cities^1^, and consequent potential for low nutritional quality these foods and drinks available in these spaces in contributing to unhealthy food choices among users exposed to this transportation^6-8^, there are few studies on the food environment of public transportation network equipment. Thus, this article aims to describe and analyze the healthiness of formal and informal food establishments in bus terminals of the MRRJ.

## METHOD

### Study design, site, and sample

A cross-sectional study was conducted between October and November 2019, a period prior to the COVID-19 pandemic. All food venues located inside or on the corresponding sidewalks of 14 bus terminals located in municipalities of the MRRJ, with a population equal to or greater than 500,000 inhabitants at the time of data collection, were included (Figure). Six municipalities met these criteria, but two terminals in the municipality of Belford Roxo were excluded due to the safety of the research team, as well as those with limited access by turnstiles. The remaining five municipalities represent 76.9% of the estimated population of the RMRJ.

**Figure:**
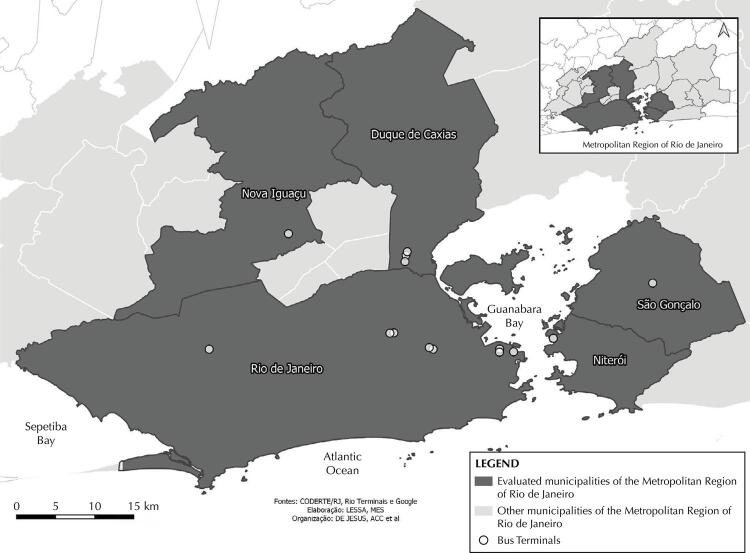
Spatial distribution of the bus terminals evaluated in the metropolitan region of Rio de Janeiro, 2019.

All commercial food establishments with a fixed point and regulated by the terminal administration were classified as formal. Unregulated commercial practice was considered as informal, including those carried out in mobile structures, such as stalls, tents, carts, or street vendors who used styrofoam boxes or hooks to display products.^13,17^.

### Assessment of the Food Environment

The formal food setting was audited using a checklist validated by Franco et al.^18^ to collect information on availability, affordability, accommodation, and promotion of food and drinks^11,12^.

The informal food setting had only the food availability dimension assessed through an inventory. To this end, an instrument was developed listing food, drinks, and culinary preparations commonly found in bus terminals within the MRRJ and providing space to insert items not foreseen in the list. From this inventory, it was possible to identify the same food subgroups used in the evaluation of formal establishments and compare the formal and informal food settings.

Data collection was carried out by a trained team of four field researchers (undergraduates) and a supervisor (postgraduate). The training was based on a field manual and consisted of four theoretical hours and four practical hours, with application of the instruments in formal and informal food establishments present on the dependencies of a public institution.

### Healthiness Indicators

The healthiness of the establishments was assessed according to the classification of food and indicators proposed by Tavares et al.^20^, which was inspired by NOVA, a classification of food that considers the extent and purpose of their industrial processing^19^. Among food, drinks or culinary preparations available in the establishments, the presence of nine selected subgroups of in natura, minimally processed or processed food and culinary preparations based on these food (INMPF) was identified, such as: fruits, raw and cooked vegetables, coconut water, etc., in addition to nine subgroups of ultra-processed food and culinary preparations containing these food (UPFP), such as packaged cookies, soft drinks, other sugary drinks, and candies. From these subgroups, four healthiness indicators related to food availability were calculated: 1) proportion of availability of INMPF subgroups among all INMPF subgroups investigated; 2) proportion of availability of UPFP subgroups among all UPFP subgroups investigated; 3) ratio of UPFP availability to INMPF availability; and 4) healthiness index, a summary measure that scores the presence of INMPF subgroups and the absence of UPFP subgroups among the investigated subgroups. The closer to 100%, the greater the healthiness of the establishments, including a bigger supply of healthy food and a lower supply of unhealthy food.

The different types of establishments were classified a posteriori according to the predominance of INMPF or UPFP indicated by the UPFP/INMPF ratio^20^: in type 1 establishments, the supply of INMPF prevailed; in type 2, there was no predominance; and in type 3, UPFP prevailed^20^. For example, when more than half of the establishments of a certain type (bonbonnières, snack bars, bars, among others) offered more UPFP than INMPF, they were classified as type 3.

### Statistical analysis

Food availability indicators were calculated for each establishment. The descriptive analysis of the formal and informal food environment of the terminals consisted of calculating means (95% confidence intervals – 95%CI) of these indicators for the set of establishments of the terminals of each municipality and for the entire MRRJ. The percentage of establishments with availability of alcoholic drinks was also calculated. The differences were considered statistically significant when the values contained in the 95%CI of the means did not overlap.

For the formal food setting, absolute and relative frequencies of the establishment characteristics were calculated, such as location, day and time of operation, type of payment, food categories with displayed advertising^18^, offering of combos and aspects that facilitate healthy choices (such as substitution of items or increase/decrease of portions). the minimum, average (standard deviation) and maximum price of each food and drink evaluated in the checklist were calculated. Prices were standardized per 100 mL or g, except for items sold at unit price.

Statistical analysis was performed in R Studio (version 4.0.3). The study was exempt from ethical review (Opinion number. 20/2019) by the Research Ethics Committee of the *Escola Nacional de Saúde Pública Sérgio Arouca* of the Fundação Oswaldo Cruz.

## RESULTS

A total of 14 bus terminals in the five most populous municipalities of the MRRJ were evaluated. The terminals had different physical structures—some had roofs (71%) and facades/identification plates (64%). They were close to commercial buildings (28%), busy roads or public transportation services (64%).

Also, 156 formal establishments were identified in the terminals, with a predominance of snack bars (46.1%), bonbonnières (24.3%), and mixed establishments (snack/coffee shops and meals) (10.9%). Informal establishments (n=127) were distributed among stalls and stands (74%), street vendors (18.1%), and carts (7.9%).

Most of the formal establishments were located in terminals of Niterói (27%), Duque de Caxias (15%), and Rio de Janeiro (14%), placed inside the terminals (73.0%), or on external sides (27.0%). Informal points of sale were more frequent in terminals of Rio de Janeiro (24%) and Niterói (21%), and inside the terminal (77.9%).

### Availability of Food

The majority of formal (95.8%) and informal (92.1%) establishments belonged to type 3 and none were classified as type 2. Only in Rio de Janeiro there were type 1 establishments (7.7%) (Table 1).

**Table 1:** Classification of formal and informal establishments in bus terminals based on the type of food predominantly sold, according to municipalities in the metropolitan region of Rio de Janeiro, Brazil (2019).

Municipality	Classification of establishments – n (%)		

	Type 1^1^	Type 3^2^	Total
Formal establishments			
Rio de Janeiro	2 (2.7)	71 (97.3)	73 (46.8)
Duque de Caxias	0 (0.0)	27 (100.0)	27 (17.3)
Nova Iguaçu	0 (0.0)	14 (100.0)	14 (9.0)
Niterói	0 (0.0)	42 (100.0)	42 (26.9)
Total	2 (1.3)	154 (98.7)	156 (100.0)
Informal points of sale			
Rio de Janeiro	9 (13.0)	60 (87)	69 (54.3)
São Gonçalo	0 (0.0)	7 (100.0)	7 (5.5)
Duque de Caxias	1 (5.0)	19 (95)	20 (15.7)
Nova Iguaçu	0 (0.0)	4 (100.0)	4 (3.1)
Niterói	0 (0.0)	27 (100.0)	27 (21.3)
Total	10 (7.9)	117 (92.1)	127 (100.0)
Total			
Rio de Janeiro	11 (7.7)	131 (92.3)	142 (50.2)
São Gonçalo	0 (0.0)	7 (100.0)	7 (2.5)
Duque de Caxias	1 (2.1)	46 (97.9)	47 (16.6)
Nova Iguaçu	0 (0.0)	18 (100.0)	18 (6.4)
Niterói	0 (0.0)	69 (100.0)	69 (24.4)
Total	12 (4.2)	271 (95.8)	283 (100.0)

^1^ Type 1: establishments providing predominantly in natura, minimally processed or processed food and culinary preparations containing such food (INMPF).

^2^ Type 3: establishments providing predominantly ultra-processed food and culinary preparations containing such food (UPFP).

The summary measure of the quality of the food environment indicated healthiness of less than 36% in the MRRJ terminals. No terminal or municipality evaluated had healthiness at least equal to 50%. Niterói presented the lowest healthiness in the MRRJ (30.6%) and São Gonçalo, the highest (41.9%). However, this municipality had only seven informal points of sale that marketed only 11.1% of the INMPF food (Table 2).

**Table 2:** Mean of healthiness indicators1 of formal establishments and informal points of sale of bus terminals, according to municipalities in the metropolitan region of Rio de Janeiro, Brazil (2019).

Municipality	Indicators – mean (95%CI)			

	Proportion of availability of INMPF^2^ subgroups	Proportion of availability of UPFP^3^ subgroups	Ratio between UPFP and INMPF availability	Healthiness index
Formal establishments				
Rio de Janeiro	26.0 (21.1–31.0)	46.7 (41.3–52.1)	2.5 (2.1–2.9)	39.6 (36.3–43.0)
Duque de Caxias	28.4 (21.4–35.4)	63.8 (55.6–72.0)	3.2 (2.3–4.0)	32.3 (28.1–3.65)
Nova Iguaçu	18.3 (8.6–27.9)	58.7 (52.9–64.6)	4.5 (3.4–5.6)	29.8 (23.8–35.7)
Niterói	15.6 (12.2–19.0)	51.3 (43.4–59.3)	4 (3.2–4.7)	32.1 (28.2–360)
Total	22.9 (20.0–25.8)	52.0 (48.3–55.7)	3.2 (2.9–3.6)	35.4 (33.3–376)
Informal points of sale				
Rio de Janeiro	9.7 (79–11.4)	35.6 (29.2–41.9)	3.9 (3.3–4.4)	37.0 (34.2–39.9)
São Gonçalo	11.1 (–)	25.4 (20.4–30.4)	2.3 (1.8–2.7)	42.9 (40.4–45.4)
Duque de Caxias	8.3 (5.5–11.2)	29.4 (19.0–39.9)	3.3 (2.2–4.4)	39.4 (34.6–44.2)
Nova Iguaçu	5.6 (-4.6–15.8)	22.2 (1.8–42.6)	2 (-10.7–14.7)	41.7 (30.2–53.1)
Niterói	11.5 (10.0–13.0)	55.1 (52.3–58.0)	4.8 (4.5–5.1)	28.2 (27.0–29.4)
Total	9.8 (8.7–10.9)	37.8 (33.6–42.0)	3.9 (3.5–4.2)	36.0 (34.1–37.9)
Total				
Rio de Janeiro	18.1 (15.1–21.0)	41.3 (37.1–45.5)	3.1 (2.8–3.5)	38.4 (36.2–40.6)
São Gonçalo	11.1 (–)	25.4 (20.4–30.4)	2.3 (1.8–2.7)	42.9 (40.3–45.4)
Duque de Caxias	19.9 (14.8-24.9)	49.2 (41.2–57.2)	3.2 ( 2.6–3.9)	35.8 (32.1–38.5)
Nova Iguaçu	15.4 (7.6–23.3)	50.6 (41.3–59.9)	4.2 (3.2–5.3)	32.4 (27.0–37.8)
Niterói	14 (1.8–1.2)	52.8 (47.9–57.7)	4.3 (3.9–4.8)	30.6 (28.2–33.0)
Total	17.0 (15.2–18.9)	45.6 (42.8–48.5)	3.5 (3.2–3.8)	35.7 (34.3–37.1)

^1^ Healthiness indicator: (total score of INMPF subgroups sold + total score of UPFP subgroups not sold)/18 × 100.

^2^ INMPF: in natura, minimally processed or processed food and culinary preparations containing such food.

^3^ UPFP: ultra-processed food and culinary preparations containing such food.

In MRRJ terminals, on average there were 250% more UPFP subgroups available for purchase than INMPF. The ratio UPFP/INMPF showed greater availability of UPFP subgroups in informal points of sale, exceeding the advantage observed in formal establishments by 70% (respectively 3.9 vs. 3.2). The average proportion of availability of INMPF in formal establishments was 22.9%; and that of UPFP was 52.0%, a higher proportion than those observed in informal points of sale (9.8% and 37.8%, respectively). Each municipality, individually, followed the same pattern, except Niterói (Table 2).

Regarding the availability of alcoholic drinks, 44.9% of formal establishments and 42.5% of informal ones sold these products, especially beers (data not shown in any table).

### Accommodations

All formal establishments operated from Monday to Friday, 72.4% on Saturdays, 32.9% on Sundays, and 18.2% on public holidays. Opening hours followed periods of greatest flow of people: 44% opened between 6am and 8am, 49% closed between 8pm and 10pm and 15.4% operated until midnight. As for payment methods, they accepted cash (100%), debit cards (78.1%), credit cards (76.1%), meal vouchers (32.9%), and digital wallets (5.2%), such as AME and Mercado Pago.

### Affordability

Regarding the prices of food sold in formal establishments, 75% could be purchased for a minimum price of up to R$ 1.00 and 83.3% for up to R$ 2.00. Regarding drinks, 46.1% and 84.6% could be purchased with the same values, respectively. Candies, desserts, French fries, fruits, fruit salad, and sandwiches had the highest average prices and price variations among the terminals. Candies, desserts, sweet cookies, packaged snacks, and ultra-processed drinks (soft drink/guaraná, iced tea/maté) were available at the lowest minimum price found among the items evaluated, less than R$ 0.80. Fruit or fruit salad had the lowest standardized price per 100 g, but high minimum and average prices when compared to the other food (Table 3).

**Table 3:** Minimum price of food and drinks sold in formal establishments of bus terminals in the metropolitan region of Rio de Janeiro, Brazil (2019).

Products	Minimum price found (Brazilian reais R$)	Average minimum price (SD) (R$)	Standardized average minimum price
Food	(R$/100 g)		
French fries	4.9	13.18 (± 8.43)	3.3
Fruit or fruit salad	2.99	6.29 (± 3.03)	1.91
Sweet or dessert	0.2	24.02 (± 29.70)	2.4
Sandwich^b^	2	5.96 (± 4.71)	- ^e^
Fried savory	0.99	3.70 (± 2.63)	- ^e^
Baked savory^b^	0.99	3.72 (± 1.75)	- ^e^
Candy (5-120 g) ^c^	0.1	1.42 (± 2.32)	3.37
Cereal bar (20-32 g)	0.99	1.45 (± 1.11)	6.51
Filled sweet cookie (20-200 g)	0.5	2.78 (± 1.90)	2.6
Sweet cookie without filling (10-200 g)	0.5	2.44 (± 3.07)	3.02
Packet snacks or savory cookies without filling (24-300 g)	0.59	2.16 (± 1.56)	2.44
Wholemeal cookie (24-300 g)	1	3.20 (± 1.39)	2.35
Drinks	R$/100 ml		
Soft drink (200-350 ml)^d^	1.49	3.46 (± 1.60)	1.11
Soft drink/natural guarana, iced tea/maté (200-450 ml)^d^	0.79	2.43 (± 1.26)	0.85
Natural fruit juice or pulp (300 ml)	1.5	5.04 (± 2.09)	1.68
Industrialized fruit juice (300 ml)	1	6.75 (± 2.98)	2.25
Nectar (350 ml)	1.19	4.85 (± 1.18)	1.38
Juice-based drink (200 ml)	1	2.81 (± 1.75)	1.4
Mineral water (200-510 ml)^d^	1	2.52 (± 0.93)	0.81
Isotonic/replenishers (500 ml)	3	6.10 (± 1.16)	1.22
Energy drinks (200-300 ml)	4	10.75 (± 2.75)	4.3
Soy-based drink (200 ml)	2	4.25 (± 3.92)	2.12
Milk or dairy-based drink (100-300 ml)^d^	1	2.72 (± 1.49)	1.7
Mixed milk and fruit drink (200-500 ml)^d^	1	4.57 (± 2.54)	1.58
Alcoholic drink (350 ml)	2	5.63 (± 1.77)	1.61

ª not considering bonbonnière item; ^b^ value per portion; ^c^ considering bonbonnière item; ^d^ according to the mean of mL. SD=standard deviation; ^e^ food without quantities in kilograms.

### Promotion

There were advertisements in 31% of formal establishments of 30 different types. Most of them referred to ultra-processed drinks (73.3%), 50% of which were sugary and 45.4% alcoholic. Desserts and ice cream were present in 20%, and only 6.6% corresponded to fresh or minimally processed food, such as fresh fruits, fruit salad, salads, natural juices, or frozen pulp preparations.

Regarding strategies related to food prices, in 20.6% of the establishments it was possible to order larger portions of food or drinks for a proportionally lower price. Only 7.7% of the establishments offered reduced portions and in 66.7% of them the prices charged were proportionally higher. In few places it was possible to make healthy substitutions at no additional cost. The exchange of French fries for salad or vegetables was available in only 12.5% of the establishments; refined rice for brown rice in 3.2%; white for whole grain bread in 2.8%; and soft drinks for natural juices, in 2.4%.

## DISCUSSION

This is believed as the first study to analyze the food environment of bus terminals in a large Brazilian urban center. We observed that different dimensions of the formal food environment and the availability of food in the informal food setting favored the consumption of ultra-processed food, which can negatively impact the diet and health of users of the public transport equipment. Although data collection occurred before the COVID-19 pandemic, it is believed that the unfavorable scenario for the consumption of healthy food has not changed in these places.

Regarding food availability, the MRRJ terminals facilitate access to UPFPs and make it difficult to INMPFs. In addition, UPFP subgroups were more competitive in terms of availability. In other words, the sale of healthy food was preferred in these spaces. In subway stations of the city of São Paulo^8^, the results were not summarized in availability indicators, but the profile of items found in formal establishments was congruent with the one observed in this study, in which more than 80% of the establishments sold at least one category of unhealthy food and 50% or more of them sold ultra-processed drinks.

The low supply of INMPF subgroups in both studies is related to the small number of type 1 establishments, such as places selling meals, and the predominance of type 3 establishments, such as snack bars and bonbonnières. This can be explained by the fact that food environments on public roads like bus terminals and subway stations, are places of passage, characterized by the offer of easily transportable food in small portions, to be consumed immediately^17^.

Thus, it is understood that MRRJ terminals are food environments that do not promote healthy food choices, as they encourage the consumption of UPFP. Thus, although this study does not refer to a geographically delimited region, the set of terminals, connected to each other, can be understood as a food swamp, due to the disproportionate exposure of its users to establishments that sell unhealthy food^21^.

While formal establishments offered a greater variety of the two food groups, informal ones presented a bigger disadvantage in offering subgroups of INMPF in relation to UPFP. This finding may be related to the simplicity of the informal sales structure^13^ and the low purchase cost of some UPFPs, as many informal vendors have low socioeconomic status, and their main source is the resale of such food^22^.

No studies were identified with specific results on informal food sales in Brazil. However, findings from a survey conducted in 2015 on buses of a public transportation company in Peru presented converging results: street vendors sold 75% of ultra-processed food, and convenience items also prevailed^7^. Street vendors play an important role in providing physically and financially accessible food, especially for people of lower socioeconomic status and working classes in large urban centers, where “mobile” eating routines can be established^17,23^. As public transportation users are lower socioeconomic status people (compared with individual transportation users)^24^, the low healthiness of establishments in the MRRJ terminals, especially in the informal points of sale, may contribute to promoting an unhealthy diet to the vulnerable portion of the population.

Additionally, we must also consider that the physical distancing measures adopted to contain the spread of COVID-19 reduced the movement of people in most of the country, at least during the first wave of the pandemic. The resulting economic crisis caused the closure of small venues and accentuated the unemployment/informality scenario within the labor market, a situation whose effects currently still persist^25,26^. Thus, the healthiness of the food environment in bus terminals may have worsened, with a greater presence of informal vendors selling food.

Regarding convenience, as in metro stations^8^, the formal establishments in MRRJ terminals present long opening hours, covering busy shifts. In addition, payment methods were diversified. The proportion of establishments that accepted meal vouchers (30%) was higher than that found in the study by Franco et al.^8^ (18.2%), although only 11% of these places offered meals. The meal voucher is a benefit granted under the Workers’ Food Program with the purpose of improving the nutrition of this population group^27^. Considering that 98.7% of formal establishments in terminals the commercialization of UPFP subgroups prevailed, the convenience of this form of payment may favor the consumption of unhealthy food, distorting the aim of the program.

As in other public transportation facilities^7,8^, the affordability of ultra-processed food and drinks was better compared to UPFP-based food and preparations. Also, the proportion of financial aspects facilitating healthy choices, such as reduced portions for a proportionally lower price or healthy substitutions at no additional cost, was low.

In Brazil, the replacement of fresh food and culinary ingredients by ultra-processed items makes food more expensive, being economically advantageous to consume meals prepared at home^28^. In MRRJ terminals, fruit or fruit salad had the lowest standardized price, but higher minimum and average prices than observed among ultra-processed items. This may affect the perception of financial advantage and lead these users to purchase unhealthy food, whose unit prices are lower.

In addition to price, marketing aspects, recognized as an important promoter of obesity, may favor the choice of unhealthy food^29^. Ads in São Paulo subway stations^8^ followed the trend observed in food availability, emphasizing ultra-processed food (65.2%) and non-alcoholic industrialized drinks (24.2%). In the MRRJ terminals, ads also promoted mostly unhealthy items: ultra-processed and alcoholic drinks.

In summary, this study showed that different dimensions of the food environment of MRRJ terminals favor the consumption of ultra-processed food. As many people use public transportation daily, these points are strategic for the promotion and sale of food and drinks. Therefore, public authorities could use these spaces to promote adequate and healthy food. The company that manages the subway stations in the city of São Paulo does not offer healthy items as a criterion for marketing food^30^. Similarly, the MRRJ terminals are managed by a state company or are under public concession^31^, which also does not establish the offer of healthy items as a rule for the concession of spaces for the formal sale of food. Informal trade, although irregular, occurs with its connivance.

This denotes the omission of the state regarding the governance of the food environment, as there is no regulation of the dynamics of ordering the spaces of terminals. Municipal governments could, for example, influence the supply and advertising of food in terminals by defining the types of establishments allowed. Similarly, contracts between public administration and private companies should have rules for the concession/supervision of food venues. Despite interventions to improve the informal food environment are more complex, one possibility would be that governments offer fiscal and logistical subsidies to registered informal vendors who sell INMPF instead of UPFP in high-traffic public places such as terminals.

As strengths, we highlight the unprecedented characterization of the food environment of terminals in a large metropolis; the census of establishments present in terminals; the description of the informal food environment; and the application of food availability indicators based on the NOVA classification.

Among the limitations, the checklist used to assess the formal food environment was adapted from an instrument for measuring the university food environment and its surroundings^18^ and was not constructed considering the NOVA classification. However, availability indicators calculated based on information from this instrument were formulated to do so^20^. Notably, that such indicators are based on the presence of food subgroups, disregarding the quantity and variety of items in each subgroup, and may underestimate the predominance of ultra-processed food. In addition, the informal food environment inside buses was not assessed; and the assessment in terminals was restricted to the availability dimension. Due to the lack of a specific instrument for this purpose, a similar instrument to the one used in the formal food environment was applied, whose validity and reliability were not studied. Nevertheless, given the scarcity of information on the informal component of the food environment and its importance in middle- and low-income countries, these limitations do not diminish the relevance of the findings, but point to the need of further research.

The results of this study lead to the conclusion that bus terminals in MRRJ do not promote healthy food choices due to the disproportionate supply of ultra-processed food in formal and informal food settings, as well as better affordability, promotion and convenience of these food in the formal environment. in the context of a large Latin American metropolitan area, where people spend much time in traffic and transportation This is a relevant finding as despite people generally stay for a little time in bus terminals, and they can become places to eat.
