# Supplementary material for: Food environment of bus terminals in the Rio de Janeiro metropolitan region
Source: Rev Saude Publica. 2024 Feb 9;58:04. doi: 10.11606/s1518-8787.2024058004769 (PMC10878687; doi:10.11606/s1518-8787.2024058004769)
Supplement: Translation

# Translation: Ambiente alimentar de terminais rodoviários da região metropolitana do Rio de Janeiro

**Authors:** Ana Carolina Castro de Jesus, Laís Vargas Botelho, Daniela Silva Canella, Letícia Ferreira Tavares, Paulo César Pereira de Castro, Isabela da Costa Gaspar da Silva, Letícia de Oliveira Cardoso

**Affiliations:** I Brasil Fundação Oswaldo Cruz. Escola Nacional de Saúde Pública Sérgio Arouca. Programa de Pós-graduação em Epidemiologia em Saúde Pública. Rio de Janeiro, RJ, Brasil.; II Brasil Universidade do Estado do Rio de Janeiro. Instituto de Nutrição. Departamento de Nutrição Aplicada. Rio de Janeiro, RJ, Brasil.; III Brasil Universidade Federal do Rio de Janeiro. Instituto de Nutrição Josué de Castro. Departamento de Gastronomia. Rio de Janeiro, RJ, Brasil.; IV Brasil Universidade Federal do Rio de Janeiro. Instituto de Nutrição Josué de Castro. Departamento de Nutrição Social e Aplicada. Rio de Janeiro, RJ, Brasil.; V Brasil Fundação Oswaldo Cruz. Escola Nacional de Saúde Pública Sérgio Arouca. Departamento de Epidemiologia e Métodos Quantitativos. Rio de Janeiro, RJ, Brasil.

**Keywords:** Alimentos, Saúde da População Urbana, Meios de Transporte

## Abstract

**OBJETIVO:**

Descrever e analisar a saudabilidade dos estabelecimentos com venda formal e informal de alimentos em terminais rodoviários da região metropolitana do Rio de Janeiro.

**MÉTODOS:**

Realizou-se auditoria em 156 estabelecimentos formais e 127 pontos informais de venda de alimentos localizados em 14 terminais rodoviários das cinco cidades mais populosas da região metropolitana do Rio de Janeiro. Foram calculadas proporções de tipos de estabelecimentos e médias (IC95%) de indicadores de disponibilidade de alimentos nos ambientes formal e informal. Para o ambiente formal, foram descritos preços, proporções das formas de pagamento aceitas, dias e horários de funcionamento e categorias de alimentos com propaganda exposta.

**RESULTADOS:**

A saudabilidade dos pontos de venda de alimentos nos terminais rodoviários era baixa (inferior a 36%). Em média, estavam disponíveis para compra 250% mais subgrupos de alimentos ultraprocessados do que *in natura* ou minimamente processados. Adquirir comida nesses locais era conveniente porque diversas formas de pagamento estavam disponíveis e os horários de funcionamento dos estabelecimentos acompanhavam os picos de movimentação. Além disso, 73,3% das propagandas se referiam a bebidas ultraprocessadas e o custo-benefício da compra de alimentos ultraprocessados era melhor que o de alimentos *in natura* ou minimamente processados.

**CONCLUSÃO:**

O ambiente alimentar dos terminais rodoviários da região metropolitana do Rio de Janeiro promove uma alimentação não saudável. Políticas públicas de regulação devem se concentrar em iniciativas que limitem a ampla disponibilidade e publicidade de alimentos ultraprocessados nesses espaços de grande circulação de pessoas.

## INTRODUÇÃO

Terminais rodoviários são pontos de conexão estratégicos entre linhas de ônibus intra e intermunicipais. Os ônibus são o meio de transporte coletivo mais utilizado nas regiões metropolitanas brasileiras, incluindo a região metropolitana do Rio de Janeiro (RMRJ)^1^, que é a segunda maior do Brasil e quarta maior da América Latina, com população estimada em 12,8 milhões de habitantes em 2019^2^.

Somente no município do Rio de Janeiro, foram registrados 1.008.326.226 embarques de ônibus em 2019^3^. Apesar de sua importância, os sistemas de mobilidade urbana são ineficientes e o tempo de viagem em grandes centros urbanos brasileiros é alto, especialmente na RMRJ, com média de 67 minutos^4^.

Estudos realizados em países de alta renda mostraram que parcela importante da alimentação fora do domicílio é realizada no caminho entre a residência e o trabalho/local de estudo^5^ e que alimentos e bebidas disponíveis no transporte coletivo têm baixa qualidade nutricional^6^. Evidências provenientes de países de baixa/média renda são concordantes sobre a baixa qualidade nutricional dos itens vendidos, mas os estudos são escassos e enfocam sobretudo a disponibilidade de alimentos^7,8^. Apesar de não ser recente o conjunto de evidências sobre como o espaço construído das cidades, áreas verdes, poluição do ar e até mesmo o ambiente alimentar impactam comportamentos individuais^9^, ainda existem lacunas quanto à caracterização do ambiente alimentar de equipamentos da rede de transporte público em metrópoles latino-americanas.

O ambiente alimentar pode ser entendido como o “contexto físico, econômico, político e sociocultural em que os consumidores interagem com o sistema alimentar para adquirir, preparar e consumir alimentos”^10^. Além da disponibilidade de alimentos e bebidas, há outras dimensões importantes para compreender a relação do ambiente alimentar com práticas alimentares: acessibilidade financeira, acomodação^11^ e promoção de alimentos e bebidas^12^.

Estudos sobre o comércio de alimentos em equipamentos da rede de transporte público priorizam abordar a venda formal^6,8^ à informal^7^. O ambiente alimentar informal é aquele não regulado por estruturas de governança formais, como quiosques e vendedores ambulantes^13^. Vendedores informais representam parcela significativa da oferta de alimentos em países de baixa e média renda, especialmente entre pessoas de baixa posição socioeconômica^14,15^. O comércio informal de alimentos e bebidas é uma prática comum em transportes públicos no Rio de Janeiro^16^.

A despeito do uso intenso do transporte público^3^, do alto tempo médio de viagem em cidades brasileiras de grande porte^1^ e do consequente potencial que alimentos e bebidas de baixa qualidade nutricional disponíveis nesses espaços têm para contribuir para escolhas alimentares não saudáveis entre os usuários expostos a esse transporte^6^, existem poucos estudos sobre o ambiente alimentar de equipamentos da rede de transporte público. Logo, este artigo tem por objetivos descrever e analisar a saudabilidade dos estabelecimentos com venda formal e informal de alimentos em terminais rodoviários da RMRJ.

## MÉTODO

### Delineamento, Local de Estudo e Amostra

Estudo transversal realizado entre outubro e novembro de 2019, período anterior à pandemia de covid-19. Foram incluídos todos os pontos de venda de alimentos situados no interior ou calçadas análogas de 14 terminais rodoviários localizados nos municípios da RMRJ, com população igual ou superior a 500 mil habitantes à época da coleta (Figura). Seis municípios preenchiam tais critérios, porém dois terminais do município de Belford Roxo foram excluídos devido à segurança da equipe, bem como aqueles com acesso limitado por catracas. Os cinco municípios restantes representam 76,9% da população estimada da RMRJ.

**Figura:** Distribuição espacial dos terminais rodoviários avaliados na região metropolitana do Rio de Janeiro, 2019.

Classificou-se como formal todo estabelecimento comercial de venda de alimentos com ponto fixo e regulamentado pela administração dos terminais. Como informal, foi considerada a prática comercial não regulamentada, realizada em estruturas móveis, como bancas, barracas, “carrocinhas” ou vendedores ambulantes que utilizavam caixas de isopor ou ganchos para expor produtos^13,17^.

### Avaliação do Ambiente Alimentar

Realizou-se auditoria do ambiente alimentar formal por meio de *checklist* validado por Franco et al.^18^para coletar informações sobre disponibilidade, acessibilidade financeira, acomodação e promoção de alimentos e bebidas^11,12^.

O ambiente alimentar informal teve somente a dimensão disponibilidade de alimentos avaliada por meio de inventário. Para tanto, foi construído um instrumento que elenca alimentos, bebidas e preparações culinárias comumente encontradas em terminais rodoviários da RMRJ e que disponibiliza espaço para inserir itens não previstos na listagem. A partir desse inventário, foi possível identificar os mesmos subgrupos alimentares utilizados na avaliação dos estabelecimentos formais e comparar os ambientes alimentares formal e informal.

A coleta de dados foi realizada por equipe treinada, composta por quatro pesquisadores de campo (graduandos) e um supervisor (pós-graduado). O treinamento, com base em um manual de campo, consistiu em quatro horas teóricas e quatro horas práticas, com aplicação dos instrumentos em estabelecimentos formais e informais de venda de alimentos presentes nas dependências de uma instituição pública.

### Indicadores de Saudabilidade

A saudabilidade dos estabelecimentos foi avaliada de acordo com a classificação de alimentos e indicadores propostos por Tavares et al.^20^, que foi inspirada na NOVA, classificação de alimentos que considera extensão e finalidade de seu processamento industrial^19^. Entre alimentos, bebidas ou preparações culinárias disponíveis nos estabelecimentos, foi identificada a presença de nove subgrupos selecionados de alimentos *in natura*, minimamente processados ou processados e preparações culinárias baseadas nesses alimentos (AIMPP), como: frutas, hortaliças cruas e cozidas, água de coco etc., além de nove subgrupos de alimentos ultraprocessados e preparações culinárias contendo esses alimentos (AUPP), como biscoitos de pacote, refrigerantes, outras bebidas açucaradas e guloseimas. A partir desses subgrupos, foram calculados quatro indicadores de saudabilidade relacionados à disponibilidade de alimentos: 1) proporção da disponibilidade de subgrupos de AIMPP entre todos os subgrupos de AIMPP investigados; 2) proporção da disponibilidade de subgrupos de AUPP entre todos os subgrupos de AUPP investigados; 3) razão entre a disponibilidade de AUPP e a disponibilidade de AIMPP; e 4) índice de saudabilidade – medida síntese que pontua a presença de subgrupos AIMPP e a ausência de subgrupos AUPP dentre os subgrupos investigados. Quanto mais próximo de 100%, maior a saudabilidade do estabelecimento, com maior oferta de alimentos saudáveis e menor oferta de alimentos não saudáveis.

Os diferentes tipos de estabelecimentos foram classificados *a posteriori* quanto à predominância de AIMPP ou AUPP indicada pela Raz-AUPP/AIMPP^20^: em estabelecimentos tipo 1, prevaleceu a oferta de AIMPP; no tipo 2, não havia predominância; e no tipo 3, predominavam AUPP^20^. Por exemplo, quando mais da metade dos estabelecimentos de certo tipo (*bonbonnières*, lanchonetes, bares, entre outros) ofertavam mais AUPP do que AIMPP, eles eram classificados como tipo 3.

### Análise Estatística

Os indicadores de disponibilidade de alimentos foram calculados para cada estabelecimento. A análise descritiva do ambiente alimentar formal e informal dos terminais consistiu no cálculo de médias (intervalos de confiança de 95% – IC95%) desses indicadores para o conjunto de estabelecimentos dos terminais de cada município e para toda a RMRJ. Também foi calculado o percentual de estabelecimentos com disponibilidade de bebidas alcoólicas. Considerou-se diferença estatisticamente significativa quando valores contidos nos IC95% das médias não se sobrepuseram.

Para o ambiente alimentar formal, foram calculadas frequências absolutas e relativas das características dos estabelecimentos, como localização, dia e horário de funcionamento, forma de pagamento, categorias de alimentos com propaganda exposta^18^, oferta de combos e aspectos facilitadores de escolhas saudáveis (como substituição de itens ou aumento/diminuição de porções). Foram calculados preço mínimo, médio (desvio-padrão) e máximo de cada alimento e bebida avaliado no *checklist.* Os preços foram padronizados por 100 mL ou g, exceto itens vendidos a preço unitário.

A análise estatística foi realizada em R Studio (versão 4.0.3). O estudo teve dispensa de análise ética (Parecer nº 20/2019) pelo Comitê de Ética em Pesquisa da Escola Nacional de Saúde Pública Sérgio Arouca da Fundação Oswaldo Cruz.

## RESULTADOS

Foram avaliados 14 terminais rodoviários, presentes nos cinco municípios mais populosos da RMRJ. Os terminais apresentavam estruturas físicas diversas entre si – alguns tinham cobertura (71%) e fachadas/placas de identificação (64%). Podiam estar próximos de prédios comerciais (28%), vias de grande movimentação ou serviços de transporte público (64%).

Foram identificados 156 estabelecimentos formais nos terminais, com predomínio de lanchonetes (46,1%), *bonbonnières* (24,3%) e estabelecimentos mistos (lanches/cafeterias e refeições) (10,9%). Os pontos de venda informal (n=127) estavam distribuídos entre barracas e bancas (74%), vendedores ambulantes (18,1%) e carrinhos (7,9%).

A maior parte dos estabelecimentos formais se encontrava nos terminais de Niterói (27%), Duque de Caxias (15%) e Rio de Janeiro (14%), localizados no interior dos terminais (73,0%) ou nas laterais externas (27,0%). Pontos de venda informal foram mais frequentes nos terminais do Rio de Janeiro (24%) e Niterói (21%), situados no interior dos terminais (77,9%).

### Disponibilidade de Alimentos

A maioria dos estabelecimentos formais (95,8%) e informais (92,1%) pertenciam ao tipo 3 e nenhum foi classificado como tipo 2. Somente no Rio de Janeiro havia estabelecimentos tipo 1 (7,7%) (Tabela 1).

**Tabela 1:** Classificação dos estabelecimentos formais e pontos de venda informal de terminais rodoviários com base no tipo de alimento predominantemente comercializado, segundo municípios da região metropolitana do Rio de Janeiro, Brasil (2019).

Município	Classificação dos estabelecimentos – n (%)		

	Tipo 1^1^	Tipo 3^2^	Total
Estabelecimentos formais			
Rio de Janeiro	2 (2,7)	71 (97,3)	73 (46,8)
Duque de Caxias	0 (0,0)	27 (100,0)	27 (17,3)
Nova Iguaçu	0 (0,0)	14 (100,0)	14 (9,0)
Niterói	0 (0,0)	42 (100,0)	42 (26,9)
Total	2 (1,3)	154 (98,7)	156 (100,0)
Pontos de venda informal			
Rio de Janeiro	9 (13,0)	60 (87)	69 (54,3)
São Gonçalo	0 (0,0)	7 (100,0)	7 (5,5)
Duque de Caxias	1 (5,0)	19 (95)	20 (15,7)
Nova Iguaçu	0 (0,0)	4 (100,0)	4 (3,1)
Niterói	0 (0,0)	27 (100,0)	27 (21,3)
Total	10 (7,9)	117 (92,1)	127 (100,0)
Total			
Rio de Janeiro	11 (7,7)	131 (92,3)	142 (50,2)
São Gonçalo	0 (0,0)	7 (100,0)	7 (2,5)
Duque de Caxias	1 (2,1)	46 (97,9)	47 (16,6)
Nova Iguaçu	0 (0,0)	18 (100,0)	18 (6,4)
Niterói	0 (0,0)	69 (100,0)	69 (24,4)
Total	12 (4,2)	271 (95,8)	283 (100,0)

^1^ Tipo 1: estabelecimentos que disponibilizam predominantemente alimentos *in natura*, minimamente processados ou processados e preparações culinárias contendo esses alimentos (AIMPP).

^2^ Tipo 3: estabelecimentos que disponibilizam predominantemente alimentos ultraprocessados e preparações culinárias contendo esses alimentos (AUPP).

A medida síntese da qualidade do ambiente alimentar indicou saudabilidade inferior a 36% nos terminais da RMRJ. Nenhum terminal ou município avaliado teve saudabilidade pelo menos igual a 50%. Niterói apresentou a menor saudabilidade da RMRJ (30,6%) e São Gonçalo, a maior (41,9%). Contudo, esse município contava com apenas sete pontos de venda informal que comercializavam somente 11,1% dos alimentos AIMPP (Tabela 2).

**Tabela 2:** Média de indicadores de saudabilidade1 de estabelecimentos formais e pontos de venda informal de terminais rodoviários, segundo municípios da região metropolitana do Rio de Janeiro, Brasil (2019).

Município	Indicadores – média (IC95%)			

	Proporção da disponibilidade de subgrupos de AIMPP^2^	Proporção da disponibilidade de subgrupos de AUPP^3^	Razão entre disponibilidade de AUPP e de AIMPP	Índice de saudabilidade
Estabelecimentos formais				
Rio de Janeiro	26,0 (21,1–31,0)	46,7 (41,3–52,1)	2,5 (2,1–2,9)	39,6 (36,3–43,0)
Duque de Caxias	28,4 (21,4–35,4)	63,8 (55,6–72,0)	3,2 (2,3–4,0)	32,3 (28,1–36,5)
Nova Iguaçu	18,3 (8,6–27,9)	58,7 (52,9–64,6)	4,5 (3,4–5,6)	29,8 (23,8–35,7)
Niterói	15,6 (12,2–19,0)	51,3 (43,4–59,3)	4 (3,2–4,7)	32,1 (28,2–36,0)
Total	22,9 (20,0–25,8)	52,0 (48,3–55,7)	3,2 (2,9–3,6)	35,4 (33,3–37,6)
Pontos de venda informal				
Rio de Janeiro	9,7 (7,9–11,4)	35,6 (29,2–41,9)	3,9 (3,3–4,4)	37,0 (34,2–39,9)
São Gonçalo	11,1 (–)	25,4 (20,4–30,4)	2,3 (1,8–2,7)	42,9 (40,4–45,4)
Duque de Caxias	8,3 (5,5–11,2)	29,4 (19,0–39,9)	3,3 (2,2–4,4)	39,4 (34,6–44,2)
Nova Iguaçu	5,6 (-4,6–15,8)	22,2 (1,8–42,6)	2 (-10,7–14,7)	41,7 (30,2–53,1)
Niterói	11,5 (10,0–13,0)	55,1 (52,3–58,0)	4,8 (4,5–5,1)	28,2 (27,0–29,4)
Total	9,8 (8,7–10,9)	37,8 (33,6–42,0)	3,9 (3,5–4,2)	36,0 (34,1–37,9)
Total				
Rio de Janeiro	18,1 (15,1–21,0)	41,3 (37,1–45,5)	3,1 (2,8–3,5)	38,4 (36,2–40,6)
São Gonçalo	11,1 (–)	25,4 (20,4–30,4)	2,3 (1,8–2,7)	42,9 (40,3–45,4)
Duque de Caxias	19,9 (14,8 -24,9)	49,2 (41,2–57,2)	3,2 ( 2,6–3,9)	35,8 (32,1–38,5)
Nova Iguaçu	15,4 (7,6–23,3)	50,6 (41,3–59,9)	4,2 (3,2–5,3)	32,4 (27,0–37,8)
Niterói	14 (11,8–16,2)	52,8 (47,9–57,7)	4,3 (3,9–4,8)	30,6 (28,2–33,0)
Total	17,0 (15,2–18,9)	45,6 (42,8–48,5)	3,5 (3,2–3,8)	35,7 (34,3–37,1)

^1^ Indicador de saudabilidade: (pontuação total de subgrupos AIMPP vendidos + pontuação total de subgrupos AUPP não vendidos)/18 × 100.

^2^ AIMPP: alimentos *in natura*, minimamente processados ou processados e preparações culinárias baseadas nesses alimentos.

^3^ AUPP: alimentos ultraprocessados e preparações culinárias contendo esses alimentos.

Nos terminais da RMRJ estavam disponíveis para compra, em média, 250% mais subgrupos AUPP do que AIMPP. A razão Raz-AUPP/AIMPP demonstrou maior disponibilidade de subgrupos AUPP nos pontos de venda informal, superando em 70% a vantagem observada nos estabelecimentos formais (3,9 vs. 3,2, respectivamente). A proporção média de disponibilidade de AIMPP nos estabelecimentos formais foi de 22,9%; e a de AUPP, 52,0% – proporções maiores do que as observadas nos pontos de venda informal (9,8% e 37,8%, respectivamente). Cada município, individualmente, seguiu o mesmo padrão, à exceção de Niterói (Tabela 2).

Quanto à disponibilidade de bebidas alcoólicas, 44,9% dos estabelecimentos formais e 42,5% dos informais vendiam tais produtos, sobretudo cervejas (dados não apresentados em tabela).

### Acomodação

Todos os estabelecimentos formais funcionavam de segunda a sexta-feira, 72,4% aos sábados, 32,9% aos domingos e 18,2% nos feriados. O horário de funcionamento acompanhava os períodos de maior fluxo de pessoas: 44% abriam entre 6h e 8h, 49% fechavam entre 20h e 22h e 15,4% funcionavam até a meia-noite. Quanto às formas de pagamento, aceitavam dinheiro (100%), cartões de débito (78,1%), crédito (76,1%), vale-refeição (32,9%) e carteiras digitais (5,2%), como AME e Mercado Pago.

### Acessibilidade Financeira

Quanto aos preços dos alimentos vendidos nos estabelecimentos formais, 75% podiam ser adquiridos por um preço mínimo de até R$ 1,00 e 83,3% por até R$ 2,00. Com relação às bebidas, 46,1% e 84,6% podiam ser compradas com os mesmos valores, respectivamente. Doces, sobremesas, batatas fritas, frutas, salada de frutas e sanduíches tiveram os maiores preços médios e variações de preços entre os terminais. Guloseimas, doces ou sobremesas, biscoitos doces, salgadinhos de pacote e bebidas ultraprocessadas (refresco/guaraná natural, chá gelado/mate) estavam disponíveis ao menor preço mínimo encontrado entre os itens avaliados, menos de R$ 0,80. Fruta ou salada de frutas apresentaram o menor preço padronizado por 100 g, mas altos preços mínimo e médio quando comparados aos demais alimentos (Tabela 3).

**Tabela 3:** Preço mínimo de alimentos e bebidas comercializados em estabelecimentos formais dos terminais rodoviários da região metropolitana do Rio de Janeiro, Brasil (2019).

Variável	Preço mínimo encontrado (R$)	Preço mínimo médio (DP) (R$)	Preço mínimo médio padronizado
Alimentos			(R$/100 g)
Batata frita	4,9	13,18 (+8,43)	3,3
Fruta ou salada de fruta	2,99	6,29 (+3,03)	1,91
Doce ou sobremesa^a^	0,2	24,02 (+29,70)	2,4
Sanduíche^b^	2	5,96 (+4,71)	-^e^
Salgado frito^b^	0,99	3,70 (+2,63)	-^e^
Salgado assado^b^	0,99	3,72 (+1,75)	-^e^
Guloseima (5–120 g)^c^	0,1	1,42 (+2,32)	3,37
Barra de cereal (20–32 g)	0,99	1,45 (+1,11)	6,51
Biscoito doce recheado (20–200 g)	0,5	2,78 (+1,90)	2,6
Biscoito doce sem recheio (10–200 g)	0,5	2,44 (+3,07)	3,02
Salgadinhos de pacote ou biscoitos salgados sem recheio (24–300 g)	0,59	2,16 (+1,56)	2,44
Biscoito integral (24–300 g)	1	3,20 (+1,39)	2,35
Bebidas			R$/100 ml
Refrigerante (200–350 ml)^d^	1,49	3,46 (+1,60)	1,11
Refresco/guaraná natural, chá gelado/mate (200–450 ml)^d^	0,79	2,43 (+1,26)	0,85
Suco de fruta natural ou polpa (300 ml)	1,5	5,04 (+2,09)	1,68
Suco de fruta industrializado (300 ml)	1	6,75 (+2,98)	2,25
Néctar (350 ml)	1,19	4,85 (+1,18)	1,38
Bebida à base de suco (200 ml)	1	2,81 (+1,75)	1,4
Água mineral (200–510 ml)^d^	1	2,52 (+0,93)	0,81
Isotônico/repositores (500 ml)	3	6,10 (+1,16)	1,22
Energéticos (200–300 ml)	4	10,75 (+2,75)	4,3
Bebida à base de soja (200 ml)	2	4,25 (+3,92)	2,12
Bebida à base de leite ou derivados (100–300 ml)^d^	1	2,72 (+1,49)	1,7
Bebida mista de leite e frutas (200–500 ml)^d^	1	4,57 (+2,54)	1,58
Bebida alcoólica (350 ml)	2	5,63 (+1,77)	1,61

ª não considerado item de *bonbonnière*; ^b^ valor por porção; ^c^ considerando item de *bonbonnière*; ^d^ segundo a média de mL. DP=desvio-padrão; ^e^ alimentos que não dispunham de quantidades em quilogramas.

### Promoção

Havia propagandas em 31% dos estabelecimentos formais, de 30 tipos diferentes. A maior parte se referia a bebidas ultraprocessadas (73,3%), sendo 50% destas açucaradas e 45,4% alcoólicas. Sobremesas e sorvetes estavam presentes em 20% e apenas 6,6% correspondiam a alimentos *in natura* ou minimamente processados, como frutas frescas, salada de frutas, saladas, sucos naturais ou preparados com polpa congelada.

Sobre as estratégias relacionadas aos preços dos alimentos, em 20,6% dos estabelecimentos era possível pedir porções maiores de alimentos ou bebidas por um preço proporcionalmente mais baixo. Apenas 7,7% dos estabelecimentos ofereciam porções reduzidas e em 66,7% deles os preços praticados eram proporcionalmente maiores. Em poucos locais era possível realizar substituições saudáveis sem custo adicional. A troca de batata frita por salada ou verduras/legumes estava disponível em somente 12,5% dos estabelecimentos; arroz refinado por integral, em 3,2%; pão branco por integral, em 2,8%; e refrigerantes por sucos naturais, em 2,4%.

## DISCUSSÃO

Acredita-se que este seja o primeiro estudo que analisou o ambiente alimentar de terminais rodoviários de um grande centro urbano brasileiro. Observou-se que diferentes dimensões do ambiente alimentar formal e a disponibilidade de alimentos no ambiente alimentar informal favoreciam o consumo de alimentos ultraprocessados, o que pode impactar negativamente a alimentação e a saúde dos usuários desse equipamento de transporte público. Apesar de a coleta de dados ter ocorrido antes da pandemia de covid-19, acredita-se que o cenário desfavorável ao consumo de alimentos saudáveis não se modificou nesses locais.

Quanto à disponibilidade de alimentos, os terminais da RMRJ facilitam o acesso aos AUPP e dificultam aos AIMPP. Além disso, subgrupos AUPP apresentavam-se mais competitivos em termos de disponibilidade. Ou seja, a venda de alimentos saudáveis era preterida nesses espaços. Nas estações de metrô da cidade de São Paulo^8^ os resultados não foram sintetizados por indicadores de disponibilidade, mas o perfil de itens encontrados em estabelecimentos formais foi congruente com o observado neste estudo, em que mais de 80% dos estabelecimentos vendiam pelo menos uma categoria de alimento não saudável e ao menos 50% deles comercializavam bebidas ultraprocessadas.

A baixa oferta de subgrupos AIMPP em ambos os estudos está relacionada ao diminuto número de estabelecimentos tipo 1, como locais que vendem refeições, e à predominância de estabelecimentos tipo 3, como lanchonetes e *bonbonnières*. Isso pode ser explicado pelo fato de ambientes alimentares de vias públicas, como terminais rodoviários e estações de metrô, serem locais de passagem, caracterizados pela oferta de alimentos facilmente transportáveis e em porções pequenas, que podem ser consumidos imediatamente^17^.

Assim, entende-se que terminais da RMRJ são ambientes alimentares que não promovem escolhas alimentares saudáveis, porque incentivam o consumo de AUPP. Nesse sentido, embora este estudo não se refira a uma região geograficamente delimitada, o conjunto de terminais, conectados entre si, pode ser compreendido como um pântano alimentar devido à exposição desproporcional de seus usuários a estabelecimentos que comercializam alimentos não saudáveis^21^.

Enquanto nos estabelecimentos formais ofertava-se maior variedade dos dois grupos de alimentos, nos informais era maior a desvantagem da oferta de subgrupos de AIMPP em relação à de AUPP. Esse achado pode estar relacionado à simplicidade da estrutura de venda informal^13^ e ao baixo custo de compra de alguns AUPP, pois muitos comerciantes informais têm baixa posição socioeconômica e sua principal fonte é a revenda desses alimentos^22^.

Não foram identificados estudos com resultados específicos da venda informal de alimentos no Brasil. Contudo, achados de uma pesquisa realizada em 2015, em ônibus de uma empresa de transporte público no Peru, foram concordantes: 75% dos alimentos comercializados por vendedores ambulantes eram ultraprocessados e itens de conveniência eram predominantes^7^. Vendedores ambulantes desempenham papel importante na provisão de alimentos acessíveis física e financeiramente, especialmente para pessoas de menor posição socioeconômica e classes trabalhadoras em grandes centros urbanos, em que podem ser estabelecidas rotinas de alimentação “móveis”^17,23^. Como usuários de transporte público são pessoas de menor posição socioeconômica (comparados a usuários de transporte individual)^24^, a baixa saudabilidade dos estabelecimentos nos terminais da RMRJ, especialmente nos pontos de venda informal, pode contribuir para promover uma alimentação não saudável à parcela vulnerável da população.

Além disso, deve-se considerar que as medidas de distanciamento físico adotadas para conter a disseminação da covid-19 reduziram a circulação de pessoas na maior parte do país, ao menos durante a primeira onda da pandemia. A crise econômica subjacente provocou o fechamento de pequenos estabelecimentos e acentuou o cenário de desemprego/informalidade no mercado de trabalho, situação cujos efeitos perduram^25,26^. Assim, a saudabilidade do ambiente alimentar dos terminais rodoviários pode ter piorado, com maior presença de vendedores informais comercializando alimentos.

Acerca da conveniência, assim como nas estações de metrô^8^, os estabelecimentos formais dos terminais da RMRJ tinham amplo horário de funcionamento, cobrindo horários de maior movimentação. Além disso, as formas de pagamento eram diversificadas. A proporção de estabelecimentos que aceitavam vale-refeição (30%) foi superior à encontrada no estudo de Franco et al.^8^ (18,2%), embora apenas 11% desses locais oferecessem refeições. O vale-refeição é um benefício concedido no âmbito do Programa de Alimentação do Trabalhador com a finalidade de melhorar a nutrição desse grupo populacional^27^. Considerando que em 98,7% dos estabelecimentos formais presentes nos terminais predominava a comercialização de subgrupos de AUPP, a conveniência dessa forma de pagamento pode favorecer o consumo de alimentos não saudáveis, o que deturpa o propósito do programa.

Assim como em outros equipamentos de transporte público^7,8^, a acessibilidade financeira a alimentos e bebidas ultraprocessados era melhor em comparação com alimentos e preparações à base de AIMPP. Ademais, foi baixa a proporção de aspectos financeiros facilitadores para escolhas saudáveis, como porções reduzidas por um preço proporcionalmente inferior ou substituições saudáveis sem custo adicional.

No Brasil, a substituição de alimentos *in natura* e ingredientes culinários por alimentos ultraprocessados encarece a alimentação, tornando-se economicamente vantajoso consumir refeições preparadas no lar^28^. Nos terminais da RMRJ, frutas ou salada de frutas tinham o menor preço padronizado, mas preços mínimo e médio mais altos do que o observado entre itens ultraprocessados. Isso pode afetar a percepção de vantagem financeira e direcionar esses usuários para a compra de alimentos não saudáveis, cujos preços unitários são mais baixos.

Além do preço, aspectos do *marketing*, que é reconhecido como um impulsionador importante de obesidade, podem favorecer a escolha de alimentos não saudáveis^29^. Propagandas presentes nas estações de metrô de São Paulo^8^ acompanhavam a tendência observada na disponibilidade de alimentos, enfatizando alimentos ultraprocessados (65,2%) e bebidas industrializadas não alcoólicas (24,2%). Nos terminais da RMRJ, as propagandas presentes também promoviam, majoritariamente, itens não saudáveis: bebidas ultraprocessadas e alcoólicas.

Sumariamente, este estudo mostrou que diferentes dimensões do ambiente alimentar dos terminais da RMRJ favorecem o consumo de alimentos ultraprocessados. Como muitas pessoas utilizam diariamente transporte público, esses pontos são estratégicos para promoção e venda de alimentos e bebidas. Logo, o poder público poderia utilizar tais espaços para promover uma alimentação adequada e saudável. A companhia que gere as estações de metrô da cidade de São Paulo não tem oferta de itens saudáveis como critério para comercializar alimentos^30^. Semelhantemente, os terminais da RMRJ são gerenciados por companhia estadual ou estão sob concessão pública^31^, a qual também não estabelece como regra a oferta de itens saudáveis para concessão de espaços destinados à venda formal de alimentos. O comércio informal, embora irregular, ocorre com sua conivência.

Isso denota a omissão do Estado quanto à governança do ambiente alimentar, pois não existe regulamentação da dinâmica de ordenação dos espaços dos terminais. Governos municipais poderiam, por exemplo, influenciar a oferta e a publicidade de alimentos nos terminais, definindo os tipos de estabelecimentos permitidos. Semelhantemente, contratos entre administração pública e empresas privadas deveriam ter regras para a concessão/fiscalização de espaços para venda de alimentos. Intervenções para melhorar o ambiente alimentar informal são mais complexas, contudo, uma possibilidade seria que os governos oferecessem subsídios fiscais e logísticos para vendedores informais cadastrados que comercializassem AIMPP em vez de AUPP em locais públicos de grande circulação, como terminais.

Como pontos fortes, destacam-se o ineditismo da caracterização do ambiente alimentar de terminais de uma grande metrópole; a realização de censo dos estabelecimentos presentes nos terminais; a descrição do ambiente alimentar informal; e a aplicação de indicadores síntese da disponibilidade de alimentos baseados na classificação NOVA.

Entre as limitações, o *checklist* utilizado para avaliar o ambiente alimentar formal foi adaptado de um instrumento para aferição do ambiente alimentar universitário e seu entorno^18^e não foi construído para considerar a classificação NOVA. Entretanto, indicadores de disponibilidade calculados com base nas informações desse instrumento foram formulados para fazer isso^20^. Ressalta-se, contudo, que tais indicadores se baseiam na presença de subgrupos de alimentos, desconsiderando a quantidade e a variedade de itens em cada subgrupo, podendo subestimar a predominância de ultraprocessados. Ainda, não foi avaliado o ambiente alimentar informal no interior dos ônibus; e nos terminais sua avaliação se restringiu à dimensão disponibilidade. Devido à inexistência de instrumento próprio para esse fim, aplicou-se instrumento dissemelhante ao utilizado no ambiente alimentar formal, cuja validade e confiabilidade não foram estudadas. Contudo, dada a escassez de informações sobre o componente informal do ambiente alimentar, e sua importância em países de média e baixa renda, tais limitações não diminuem a relevância dos achados, mas apontam a necessidade de mais pesquisas.

Os resultados deste estudo permitem concluir que terminais rodoviários na RMRJ não promovem escolhas alimentares saudáveis devido à oferta desproporcional de alimentos ultraprocessados nos ambientes alimentares formal e informal, bem como pela melhor acessibilidade financeira, promoção e conveniência desses alimentos no ambiente formal. Esse é um achado relevante no contexto de uma grande região metropolitana latino-americana, onde pessoas passam mais tempo no trânsito e nos meios de transporte, de modo que pontos de conexão, como terminais rodoviários, embora sejam locais de passagem rápida, podem se tornar locais de realização de refeições.
